# A Bacteria and Cell Repellent Zwitterionic Polymer Coating on Titanium Base Substrates towards Smart Implant Devices

**DOI:** 10.3390/polym13152472

**Published:** 2021-07-27

**Authors:** Mona Es-Souni, Martha Es-Souni, Hamzah Bakhti, Aydin Gülses, Helge Fischer-Brandies, Yahya Açil, Jörg Wiltfang, Christian Flörke

**Affiliations:** 1 Department of Oral and Maxillofacial Surgery, Faculty of Dentistry, CAU, 24103 Kiel, Germany; monaesouni@gmail.com (M.E.-S.); aydin.guelses@uksh.de (A.G.); yahya.acil@uksh.de (Y.A.); Joerg.wiltfang@uksh.de (J.W.); christian.floerke@uksh.de (C.F.); 2 Department of Orthodontics, Faculty of Dentistry, CAU, 24103 Kiel, Germany; helge.fischer-brandies@uksh.de; 3 Department of Mathematics, University of Hamburg, 20146 Hamburg, Germany; Hamzah.bakhti@uni-hamburg.de

**Keywords:** zwitterionic polymer coating, photopolymerization, antibiofouling, cytotoxicity, titanium substrate

## Abstract

Biofouling and biofilm formation on implant surfaces are serious issues that more than often lead to inflammatory reactions and the necessity of lengthy post-operation treatments or the removal of the implant, thus entailing a protracted healing process. This issue may be tackled with a biocompatible polymeric coating that at the same time prevents biofouling. In this work, oxygen plasma-activated silanized titanium substrates are coated with poly(sulfobetaine methacrylate), a zwitterionic antibiofouling polymer, using photopolymerization. The characterization of polymer films includes FT-IR, AFM, and adhesion strength measurements, where adhesion strength is analyzed using a cylindrical flat punch indenter and water contact angle (WCA) measurements. Both cytotoxicity analysis with primary human fibroblasts and fluorescence microscopy with fibroblasts and plaque bacteria are also performed is this work, with each procedure including seeding on coated and control surfaces. The film morphology obtained by the AFM shows a fine structure akin to nanoropes. The coatings can resist ultrasonic and sterilization treatments. The adhesion strength properties substantially increase when the films are soaked in 0.51 M of NaCl prior to testing when compared to deionized water. The coatings are superhydrophilic with a WCA of 10° that increases to 15° after dry aging. The viability of fibroblasts in the presence of coated substrates is comparable to that of bare titanium. When in direct contact with fibroblasts or bacteria, marginal adhesion for both species occurs on coating imperfections. Because photopolymerization can easily be adapted to surface patterning, smart devices that promote both osseointegration (in non-coated areas) and prevent cell overgrowth and biofilm formation (in coated areas) demonstrate practical potential.

## 1. Introduction

Antibiofouling coatings based on polymer brushes are of paramount importance in various biomedical and biotechnological applications. They afford an environmentally benign and sustainable way to preventing the adhesion of proteins and different cell types [1,2,3]. Among the plethora of materials available, zwitterionic brushes are the most promising as most of them combine biocompatibility with protein and cell repellent properties. Furthermore, they can be used with various substrates, including metals, ceramics, and polymers, using well-known grafting techniques [3,4,5,6,7,8]. The mechanisms governing the antibiofouling properties of these polymers have been amply discussed in the relevant literature [3,5,9]. It appears that their high hydrophilicity leads to a watery surface (a hydrated layer that has been proven to be non-structured, i.e., the water hydrogen bonds are not perturbed) that prevents adsorption as no free energy is gained by the adsorption of the protein on the watery surface. Furthermore, steric effects and surface neutrality that precludes ion exchange also seem to relate to the main factors inhibiting protein adsorption [3].

Titanium base alloys are state-of-the-art implant materials in various surgery applications, including dentistry, bone and craniofacial reconstruction/fixation [10,11], and temporary anchorage devices (TAD), etc. Titanium is a lightweight and bioinert metal that possesses a high specific strength (strength to density ratio) and an elastic modulus close to that of human bone. Titanium additionally features excellent corrosion resistance and relatively high X-ray translucency, thus facilitating post-treatment diagnosis [10,12]. Titanium is known to promote osseointegration, which is desirable in most cases of dental and orthopedic reconstructive surgeries. Nevertheless, implant removal is indicated in case of complications, including “infections, non-union, failure of fixation, pain after fracture consolidation, etc.” [13]. The literature also contains reports on complications related to TADs, such as periimplantitis, inflammations, and cell overgrowth [14]. A surface treatment that could prevent bacteria and cell adhesion while maintaining biocompatibility is consequently highly desirable in many cases. Various strategies have been devised to endow biomaterial surfaces with anti-biofouling properties [3,4,5,6,7,15,16,17,18]. Zwitterionic polymer films appear to be among the most promising strategies as they fulfill the required functionality with simultaneous proven biocompatibility [3]. In recent works [19,20], it has been shown that polySBMA (poly(sulfobetaine methacrylate)) can be successfully grafted from/onto the pore walls of porous aluminum and titanium oxide films (obtained via anodization of the metal) using a versatile and environmentally friendly photografting method. The polySBMA films were shown to wet the pore walls, leading to the creation of a 3D nanocomposite that was mechanically resistant and particularly effective at repelling proteins and bacteria. Furthermore, the same authors demonstrated that it is possible to structure the surface of the substrate in neighboring areas in order to achieve high protein adhesion (non-coated with polySBMA) and high protein repellant (coated with polySBMA) effects. As such, the authors demonstrated that it is possible to endow a particular implant with areas of high cell adhesion where cell adhesion is desirable, e.g., for osseointegration, and areas with poor or even no cell adhesion where such functionality is required.

In the present work, titanium sheets of a commercial purity are coated with a layer of the zwitterionic polymer polySBMA using a photographing technique. Emphasis is placed on systematic assessment of cytocompatibility and microbial adhesion; however, structural investigations and analyses of the wetting and nanomechanical properties are also succinctly reported in order to sketch a nearly complete account of the properties of the film. With respect to the nanomechanical properties, the coating adhesion strength after storing in water and an aqueous solution of 3% NaCl is reported. It is shown here that the human gingival fibroblast and dental plaque adhesion on the processed coatings is almost negligible; however, this does not represent a lack of biocompatibility, as the cytocompatibility is not different from that of bare substrates. With respect to the nanomechanical properties, the high adhesion of the coatings is demonstrated and seems to be boosted when stored in NaCl before testing.

## 2. Materials and Methods

### 2.1. Chemical Substances, Culture Vessels, and Explant Material

The chemical substances used in this work were the following: 3-(trimethoxysilyl)propyl methacrylate (TMSPMA, Sigma Aldrich, St. Louis, MI, USA); [2-(methacryloyloxy)ethyl]dimethyl-(3-sulfopropyl)ammonium hydroxide, 95% (SBMA, Sigma Aldrich); 1-phenyl-1,2-propanedione (PPD, Sigma Aldrich); ethanol, 99.9% (Walter CMP, Kiel, Germany); double-distilled water (Carl Roth, Germany); 10 mM PBS (*w/o* Ca, Mg, Biowest); Penicillin-Streptomycin 100X (pen. 60 mg/L and strep. 100 mg/L, Biowest); amphotericin B 100X (AmB 250 mg/L, Biowest); fetal bovine serum (FBS, BioSell); alpha-modified minimum essential medium (α-MEM, Sigma, Tokyo, Japan); RPMI1640 (RPMI, Biowest); sodium 3,3′-{-[(phenylamino)carbonyl]-3,4-tetrazolium}-Bis(4-methoxy-6-nitro)benzenesulfonic acid hydrate (XTT, Serva); methylphenazinium methylsulfate (PMS, Serva); 4′,6-diamidin-2-phenylindol (DAPI, Roth); propidium iodide (PI, Serva); methanol, >99.9% (Sigma); CASO Bouillon (Chemsolut); Columbia agar +5% sheep blood (SBA, Biomerieux, Marcy-lÉtoile, France); 0.9% NaCl (NaCl, Fresenius Kabi).

All culture vessels and inserts used were of a tissue culture (TC) quality (Sarsted). Nunc Thermanox coverslips of a 13 mm diameter were obtained from Thermo Scientific.

For cell cultures of primary human gingival fibroblasts (hgF), explant material from a retracted molar of a healthy donor was used. The donor’s written informed consent was obtained before the surgical intervention.

### 2.2. Coating Procedure

Commercially pure grade 1 titanium (TiCP) sheets were purchased in an annealed, oxide scale-free, and straightened condition from Goodfellow (0.1 mm in thickness; Goodfellow Germany). The sheets were cut into 0.75 × 1 cm^2^ samples, degreased in 9.9% ethanol in an ultrasonic bath, rinsed twice with ethanol, and then dried with compressed air. These substrates were then treated with oxygen plasma (300 W; 0.2 mbar O_2_ pressure; Plasma Technology, Herrenberg Gültstein, Germany) for 5 min and 30 s. Subsequent to this activation treatment, the substrates were immediately primed with TMSPMA using a chemical vapor deposition process as described in previous articles [19,20]. Coating onto the primed substrate surfaces with polySBMA was conducted using a photopolymerization process. For this purpose, two solutions were separately prepared, namely an aqueous solution containing 28% (*w/w*) SBMA and a 52 mM PPD initiator solution in 2-propanol. After degassing of the monomer solution, the initiator was added in a molar ratio of 1 PPD:62.5 SBMA (volume ratio 1:3). Subsequently the samples were placed in a PTFE holder containing round cavities (15 mm in diameter and 2 mm in depth) and each was covered with 0.35 mL of the SBMA/PPD-solution. Finally, in order to minimize the oxygen inhibition effects during polymerization, the samples were covered with a borosilicate glass (0.75 mm in thickness) and were treated with UV irradiation at 360 nm for 4 min. Five subsequent rinsing steps (20 s of vortex mixing for each in H_2_O) and a final ultrasonic cleaning step in H_2_O (20 s) were adapted to eliminate non-polymerized and loosely bound monomers. A drying procedure with compressed air finished the coating process. The minimum thickness of the film was estimated from indentation measurements to be in the range of 250 nm (from indentation depth curves, where the modulus and hardness of the film on the Ti-substrate were characterized (not mentioned in this paper)). Ellipsometry measurements could not be performed because of the high roughness of the substrate surface that was used as received (see microscopic images in Figure 2 below).

### 2.3. Structural and Morphological Characterization

The structure of the polymer layer was assessed using an attenuated total reflection-Fourier transform infrared spectrometer (ATR-FTIR) produced by Perkin Elmer (Waltham, MA, USA). FTIR spectra were recorded between 4000–400 cm^−1^.

The topographies and microstructures of the films were investigated using a high-resolution scanning electron microscope (SEM) (Ultra Plus, Zeiss, Oberkochen, Germany) equipped with an energy dispersive X-ray spectroscopy (EDS) package (INCAx-act, Oxford Instruments, Abingdon, UK). Furthermore, the fine structures of the surfaces were investigated at a high resolution using atomic force microscopy (AFM, Nanowizard, JPK, Berlin, Germany). Measurements were taken in the dry condition.

The wettability of coatings was measured with a water contact angle instrument (Data Physics Instruments GmbH, Filderstadt, Germany) using the sessile drop method. Non-grafted samples were also measured for comparison. For each sample, the water contact angle (WCA) was measured in three different positions and the mean values are reported here.

### 2.4. Nanomechanical Characterization of the Adhesion Strength

The nanomechanical characterization of the adhesion strength of the polySBMA coatings was conducted using iMicro^®^ machine for nanoindentation and nanoscratch testing (Nanomechanics Inc., KLA Tencor, Oak Ridge, TN, USA) that was mounted on an adaptive stage for the purpose of vibration damping. A flat punch cylindrical indenter with a nominal diameter of 100 μm (107.75 μm and 107.72 μm vertically and horizontally, respectively) was used for this purpose. The flat punch indenter was brought into contact with the polySBMA coating with a maximum load of 2 mN. The pull-in and pull-off forces, as well as the penetration depth, were recorded continuously during loading and unloading.

Using the data obtained from the nanoindentation depth–load curves, the pull-off stress, noted as $\sigma_{\mathrm{pull}-\mathrm{off}}$, is equal to the maximum pull-off force, $P_{\mathrm{pull}-\mathrm{off}}$, per cross-sectional area of the flat punch indenter, $A={\pi D}^{2}/4$, namely:

(1) $$\sigma_{\mathrm{pull}-\mathrm{off}}=4\frac{P_{\mathrm{pull}-\mathrm{off}}}{{\pi D}^{2}}\;$$

where D=100 μm is the diameter of the cylindrical end of the flat punch tip.

### 2.5. Interaction of Substrates with Cell and Bacteria Cultures

#### 2.5.1. Cytocompatibility

Prior to biological tests, the samples were first immersed in 70% ethanol, dried, and then left overnight on a sterile bench under an UV light. HgF cultures prepared in an alpha-modified minimum essential medium (α-MEM, Sigma) supplemented with 10% FBS (BioSell) and antibiotics were prepared, as described in [21]. Cells from the 7th to 15th passages were used for quantitative cytocompatibility testing and qualitative cell growth detection. The evaluation of the in vitro cytocompatibility of coated samples compared to that of the samples in the received state followed the specifications of the international standard ISO 10993-5:2009E. In brief, 2 × 10^4^/0.75 mL α-MEM/well was seeded in 12-well plates and allowed to settle for 1 h. Subsequently TC inserts containing the samples facing the cell culture were added to each well and the volume of the medium was raised to 1.2 mL/well in order to ensure coverage of the whole sample. Each plate contained 3 coated samples, 3 bare samples, 3 empty inserts, and 3 blanks. Incubation periods of 24, 48, and 72 h were chosen (37.5 °C; 5% CO_2_; 95% RH). At the end of each incubation, the inserts and used media were discarded and replaced with 0.28 mM XTT/2 μM PMS dissolved in a phenol red-free RPMI-1640 medium supplemented with 10% FBS. The reduction of the almost colorless tetrazolium salt to the orange formazan product, driven by metabolically active cells, was allowed to proceed for 3 h. After the 3 h, 6 measurements/well (150 µL) at an optical density of 470/750 nm (µQuant microplate spectrophotometer, BioTek Instruments) were taken and averaged. An average was assigned to a single sample. Table 1 specifies the sums of samples/types/incubation periods.

The cell-repellent properties of the polySBMA coating were assessed in a qualitative manner by bright field (BF) and fluorescence microscopy (Motic AE31E inverse microscope equipped with a digital camera (Toupcam, UCMOS), a fluorescence DAPI/Hoechst/AlexaFluor 350 filter set, and the Toup View software package). PolySBMA-coated and bare Ti specimens, as well as coverslips, were placed in 12-well TC plates und subsequently covered with a 5 × 10^4^ hgF/0.75 mL supplemented α-MEM/well. After an incubation of 72 h at 37.5 °C with 5% CO_2_ and 95% RH, media were discarded, and the cultures were washed with PBS and finally incubated for 20 min in a methanolic 14.2 µM DAPI solution at room temperature (RT, orbital shaker). Subsequently, the cultures were rinsed once with methanol and twice with PBS. For fluorescence microscopy, each sample was positioned with the cell culture side facing the incident angle of the UV light.

#### 2.5.2. Plaque Culture

Plaque from a healthy donor was taken with a sterile cotton swap from their dental arches. The plaque samples were suspended in 2 mL of CASO and 40 µL of the thoroughly vortexed suspension was plated on SBA and incubated for 18 h at 37 °C. The complete layer of germs was then suspended in CASO to a final concentration of 0.093 OD_660nm_/200 µL, corresponding to approximately 6 × 10^7^ germs/mL. Following this, 350 µL of this suspension was used to cover the coated, bare, and coverslip samples placed in 24-well TC plates for an incubation period of 72 h (37 °C with agitation). After every 24 h, 350 µL of fresh CASO was added to each well. Following the incubation, each sample was vortexed and rinsed three times in 30 mL of sterile water and stored when dry for SEM analysis or in sterile 0.9% NaCl for fluorescence microscopy. In the latter case, the samples were covered with 0.5 mL of 6 µM PI in NaCl for 2 min at RT, then rinsed and positioned with the culture side facing the incident angle of the UV light.

### 2.6. Statistical Analysis

Statistical analysis was carried out using the Origin 8 data analysis and graphing software package (OriginLab corporation, Northampton, MA, USA). One-way ANOVA tests were conducted to evaluate the differences between means. Tukey’s post-hoc test was used in cases of significantly different mean values. The significance level was set to *p* < 0.05 here.

## 3. Results

### 3.1. Structural and Morphological Characterization

#### 3.1.1. FTIR Investigations

**Figure 1:**
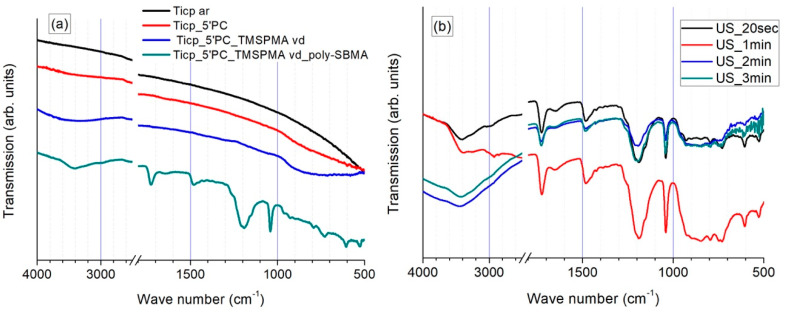
(**a**) ATR-FTIR spectra from differently treated TiCP samples. (**a**,**b**) PolySBMA coating after cumulative ultrasonic treatment from 20 s to 3 min (US). TiCp ar: as-received titanium substrate; TiCP 5’PC: after 5 min of oxygen plasma treatment; TiCP 5’PC_TMSPMA vd: vapor deposited TMSPMA primer; TiCP 5’PC_TMSPMA vd_polySBMA: photopolymerized SBMA.

The characterization of specific chemical bonds within molecules located on the surfaces of bare, plasma-activated, primed, and polySBMA coated samples was performed by means of ATR-FTIR spectroscopy in the range of 4000–400 cm^−1^. Representative transmission spectra are shown in Figure 1 and the major vibration bands are summarized in Table 2.

As depicted in Figure 1 and Table 2, the FTIR spectra of the samples in different states of treatment presented easily distinguishable features. A slight surface hydroxylation of TiCP samples right after O_2_ plasma activation was detected, while TiCP in the as-received state only showed a featureless transmission spectrum. The presence of silanol and siloxane species on the TMSPMA-primed surfaces was confirmed by the broad bands in the corresponding regions; however, they featured low intensities due to the extremely low thickness of the primer layer. In contrast, the polySBMA-coated samples presented intense vibration bands, and the typical stretching vibrations around 1725 and 1180/1040 cm^−1^ can be attributed to the presence of carbonyl and sulfonate groups, respectively. Furthermore, the vibrations around 3400 and 1640 cm^−1^ may be assigned to physisorbed moisture.

It must be pointed out that the FTIR spectra of the polySBMA coated samples were still present even after 3 min of sonication in deionized water (Figure 1b). This attests to the high adhesion strength of the coating and its resistance to cavitation (see below for the adhesion strength of the coating determined via nanomechanical characterization).

#### 3.1.2. Topography and Microstructure

**Figure 2:**
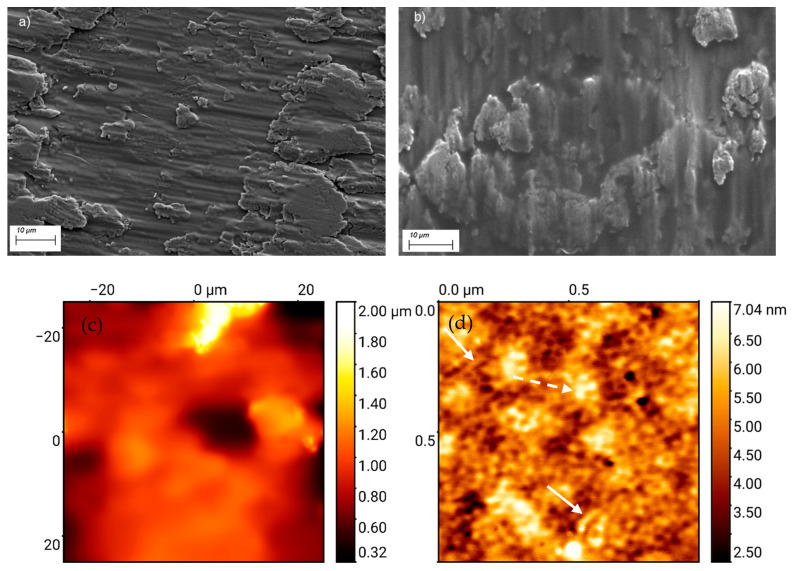
(**a**) Secondary electron (SE) micrograph of the plasma-cleaned TiCP substrate surface. (**b**) SE micrograph of the same surface after the polySBMA coating procedure. The surface topography is not substantially affected by the coating. (**c**) AFM amplitude micrograph of the polySBMA film on Ti, showing a largely featureless structure that demonstrates the underlying substrate roughness. (**d**) AFM amplitude micrograph at a higher resolution. The micrograph suggests nanoropes of tethered polymer brushes either laying in the plane of the surface (plain arrows) or agglomerating in domains of larger bundles (dotted arrows).

Secondary electron (SE) micrographs of plasma-treated and polySBMA-coated TiCP surfaces are shown in Figure 2a,b. As can be seen, the rough topography of the surface was marginally affected by the coating. AFM examination of the coated sample at low magnification shows a featureless morphology with polySBMA replicating the original roughness of the surface (Figure 2c) in accordance with the SE micrographs; however, at a higher resolution, the nanostructured morphology of the film is revealed. Figure 2d suggests that the film consists of nanoropes that are probably tethered bundles of polymer brushes that are either laying in the plane of the surface or agglomerating in domains of larger bundles that protrude perpendicular to the surface; however, the surface roughness remains very low at this magnification (see the side bar). This particular intertwined nanorope structure may conveniently explain the high mechanical resistance of the film mentioned above. It should be pointed out that AFM studies of zwitterionic films with similar morphologies have not yet been reported in the literature. Presumably, the depicted morphology arises from the electrostatic interaction between polymer brushes, resulting in “an ionically cross-linked network structure” [8].

### 3.2. Nanomechanical Adhesion Strength

**Figure 3:**
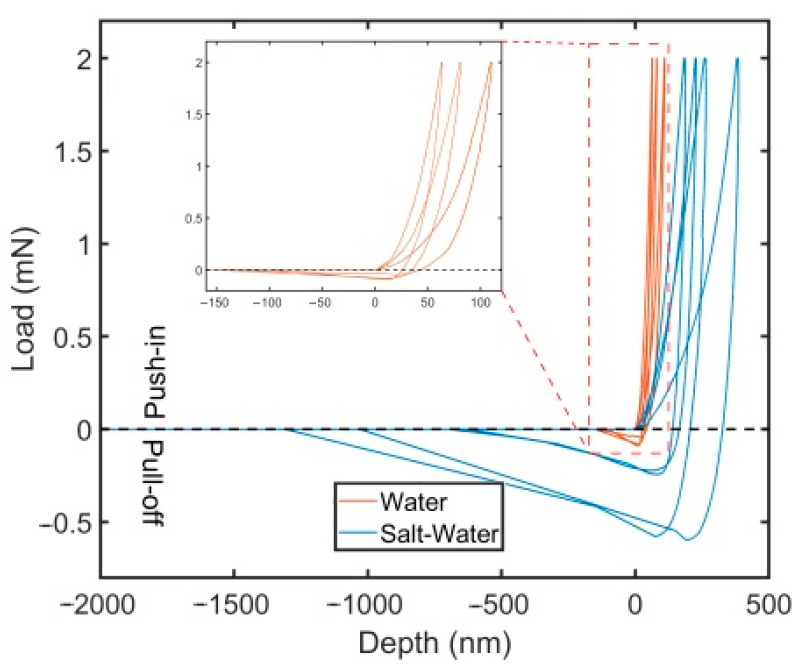
Nanoindentation depth–load curves obtained from the cylindrical flat punch adhesion testing for the polySBMA coatings soaked in water and saltwater. The inset shows a magnification of the results in deionized water.

The nanoindentation adhesion testing was conducted for the polySBMA coating after soaking in deionized water or 0.51 M of NaCl (3% NaCl) for one day. The measurements of the depth–load curves for each case are presented in Figure 3, which depicts multiple tests for different samples. There are noticeable differences between the two cases, for instance, both the maximum depth reached before pull-off and the travel distance of the flat punch before reaching the point of zero load were much larger in saltwater. Because the product of depth (load) and travel distance is only rough measure of the adhesion work, soaking the films in saltwater results in a substantially higher interfacial adhesion strength between the polymer coating and the flat punch indenter.

The results obtained using Equation (1) are shown in Table 3, where the mean values of maximum depth and pull-off force were extracted from the recorded depth–load curves for a maximum load of 2 mN. This finding, which has been confirmed for polySBMA films on two additional substrates (report in preparation), provides a description of a method to impart better adhesion properties for polySBMA coatings with the flat punch indenter via soaking for a short time in saltwater (3% NaCl). To the best of our knowledge, a similar finding has not yet been reported. It is well known that polySBMA is not soluble in water [8], where only marginal swelling has been observed, but some degree of loosening of the inter/intra chain network has been reported for NaCl solutions, particularly at low NaCl concentrations, i.e., <0.5 M [22,23,24,25], essentially due to the binding of the salt ions to zwitterions, thus weakening the electrostatic intra/inter-zwitterionic chain attraction forces. This results in strong swelling and consequently in a strong hydration of the polymer. Similar results with a higher adhesion strength have been reported for PDMS films, although swelling was performed with PDMS-free chains for these films [26]. We surmise that the swelling of the polySBMA film in the NaCl solution results in larger contact area of the polymer brushes with the flat-punch indenter, and thus resulting in a higher adhesion strength; however, the factors influencing the interfacial adhesion of polySBMA with the primed substrate required further in-depth investigation. At present, it can only be stated that the film integrity was not affected after soaking in 0.51 M NaCl nor after the biocompatibility/biofouling studies below, which were all conducted for times ranging between 24 and 72 h in cell nutrition media that contained a number of salts in typical physiological concentrations.

### 3.3. Wetting Properties

**Figure 4:**
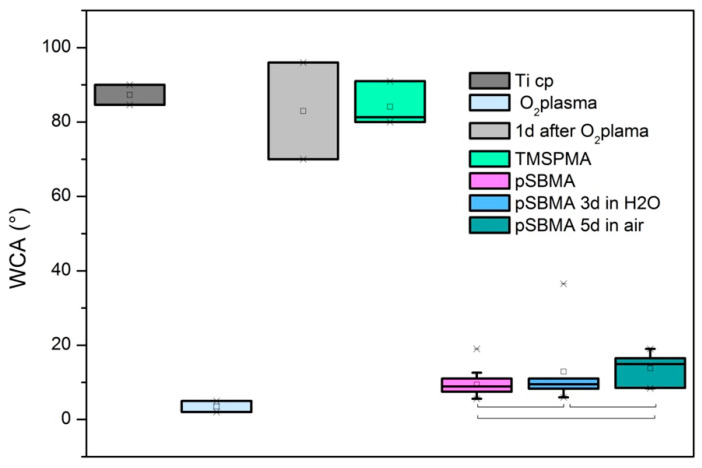
Comparative water contact angle (WCA) measurements of the different surfaces: TiCP: as-received TiCP; O_2_ plasma: TiCP after oxygen plasma treatment; 1 d O_2_ Plasma: TiCP aged for 24 h after plasma treatment; TMSPMA: TiCP with O_2_ plasma treatment followed by a CVD treatment with TMSPMA; pSBMA: TMSPMA-primed surface followed by photografting with polySBMA; pSBMA for 3 d in H_2_O: polySBMA-coated surface soaked in deionized water for 72 h; pSBMA for 5 d in air: pSBMA with dry aging for 5 days. Statistical analysis was performed on the polySBMA coated surface using ANOVA testing.

The water contact angle (WCA) measurements corresponding to different surface treatment/ageing are displayed in Figure 4. The as-received surface showed a high WCA that approached 90°. Such an angle could arise from the adsorption of carbonaceous impurities. After oxygen plasma treatment, the surface became superhydrophilic with a WCA ≤ 5°, but when this surface was aged 24 h in air it flipped back to the original WCA. The primer treatment (TMSPMA) directly applied after plasma activation presented a resulting WCA in the range of 90°. This WCA arises from the hydrophobic nature of the propyl/methyl groups of TMSPMA. After coating with polySBMA, a nearly superhydrophilic surface was obtained. The WCA of this surface increased slightly after 5 days of dry storage in air; however, the sample retained superhydrophilic properties, which attests to the stability of the polySBMA coatings. Table 4 summarizes the results obtained, along with their mean standard deviations. In order to show the stability of the results of the polySBMA coated samples, they were compared using one-way ANOVA testing. The results are depicted in Figure 4. At the 0.05 significance level, the population means were not significantly different (overall ANOVA: F(2.29) = 3.15; *p* = 0.05766).

### 3.4. Cellular Interaction with Poly SBMA Coated TiCP Samples

#### 3.4.1. XTT Viability Testing and Microscopy

**Figure 5:**
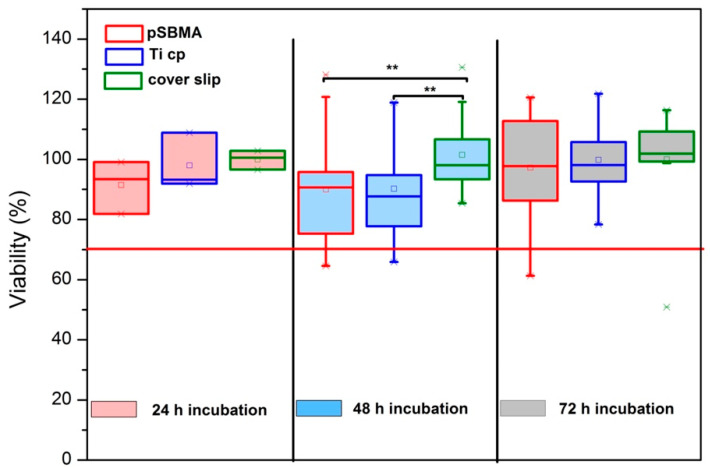
Viability results of hgF-facing inserts containing pSBMA-coated TiCP (pSBMA), bare TiCP (TiCP), and Thermanox cover slips for different incubation periods: 24 h (red), 48 h (blue), and 72 h (gray). A threshold of 70% for the cytotoxic potential of materials (ISO 10993-5:2009E) is indicated by the red line. One-way ANOVA testing was used to compare the viabilities obtained for the presence of different sample surfaces within each incubation period. ** denotes significantly different results obtained via a Tukey post-hoc test.

Figure 5 presents a box plot chart of the viability rate of hgF in indirect contact with bare TiCP, polySBMA-coated samples, and Thermanox cover slips for 3 different incubation times. One-way ANOVA testing was used to compare the viabilities obtained within each group. At the 0.05 significance level, there was no significant difference in the viability found within groups during the 24 h and 72 h of incubation (24h: F(2.6) = 1.019; *p* = 0.416/72 h: F(2.57) = 0.238; *p* = 0.789). There was a statistically significant difference in the viability of the 48 h incubation period at the 0.05 significance level (F(2.57) = 3.962; *p* = 0.0245). A Tukey post-hoc test performed with this group revealed a significant difference between the Thermanox control and (1) polySBMA coated titanium (*p* = 0.0443), as well as (2) TiCP (*p* = 0.0487). In contrast, the coated and bare samples showed no significantly different viabilities (*p* = 0.999), leading to the overall conclusion that the coated samples exhibit comparable cytocompatibility to bare titanium within the test conditions considered here.

**Figure 6:**
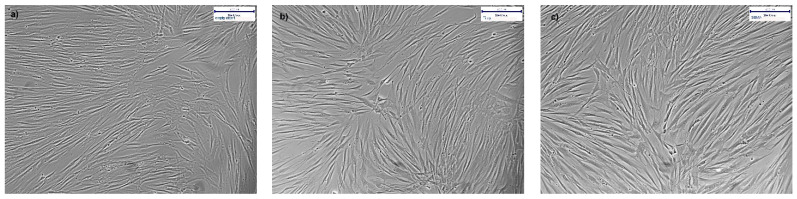
(**a**–**c**): 72 h hgF culture in indirect contact with a Thermanox cover slip (**a**), TiCP (**b**), and polySBMA-coated TiCP (**c**).

The results were confirmed by bright field microscopy. The micrographs of morphologically intact cultures with hgF for 72 h in indirect contact with Thermanox cover slip, TiCP, and polySBMA-coated TiCP in Figure 6a–c confirm the cytocompatibility of the coating.

#### 3.4.2. Proliferation of hgF on PolySBMA Coated TiCP

**Figure 7:**
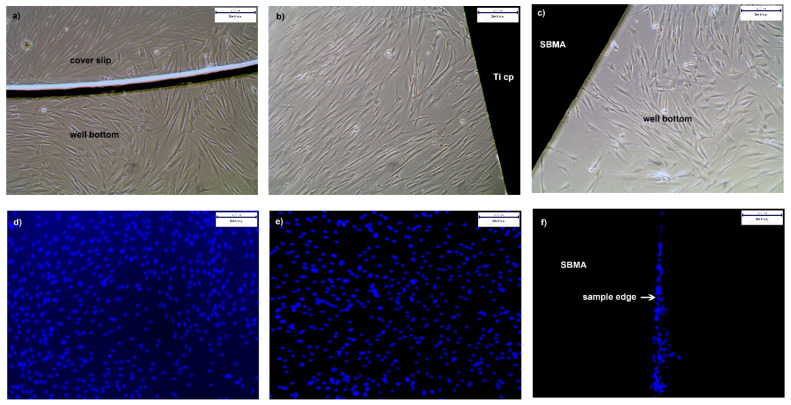
hgF in direct contact with samples. (**a**–**c**) Bright field and (**d**–**f**) fluorescence micrographs of hgF seeded in the wells containing coverslips (**a**,**d**), as-received TiCP (**b**,**e**), and polySBMA coatings (**c**,**f**) after 72 h of incubation. Notice the tiny string of hgF that adheres solely to the edge of the polySBMA-coated sample (arrow in (**f**)).

Cell adhesion and proliferation of hgF on coated TiCP, in comparison to the bare TiCP and coverslips, was assessed qualitatively via bright field and fluorescence microscopy. Figure 7a–c depict a dense proliferation of morphologically intact hgF around the coverslips, TiCP, and SBMA-coated substrates. This is in good agreement with the results of indirect contact assays. Figure 7d–f finally present the situation on top of the different surfaces studied. As can be seen, cell proliferation visualized by DAPI nucleus staining of adherent hgF presents a dense configuration on coverslips and TiCP (ar) surfaces. Intact SBMA coatings appear to prevent hgF adhesion, in contrast to coating defects such as deep scratches or edges where shallow strings of adherent hgF can be clearly seen (edges are difficult to coat because of solution dewetting at sharp edges and/or non-accessibility of the UV radiation to hidden edges).

#### 3.4.3. Cultures from Plaque Samples

**Figure 8:**
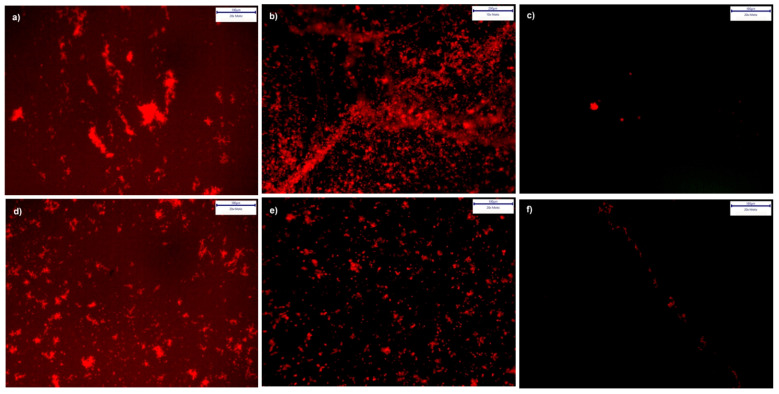
Biofilm formation on specimen surfaces: (**a**–**f**) show fluorescence micrographs of PI-stained plaque cultures after 72 h incubation on coverslips (**a**,**d**), TiCP (ar) (**b**,**e**) and polySBMA coatings (**c**,**f**).

The interactions of PI-stained cultures originating from dental plaque with the samples are depicted in Figure 8a–f. PI is often used in microbial cell viability assays. The stain passes through cell membrane defects of injured (reversibly damaged membranes [27]), or dead cells and intercalates with cellular double-stranded DNA. This method allows aggregates of damaged adherentncells to be visualized on the sample surface [28]. For each specimen, micrographs of two image sections are displayed in Figure 8 to demonstrate the degrees of biofilm formation after 72 h of incubation.

**Figure 9:**
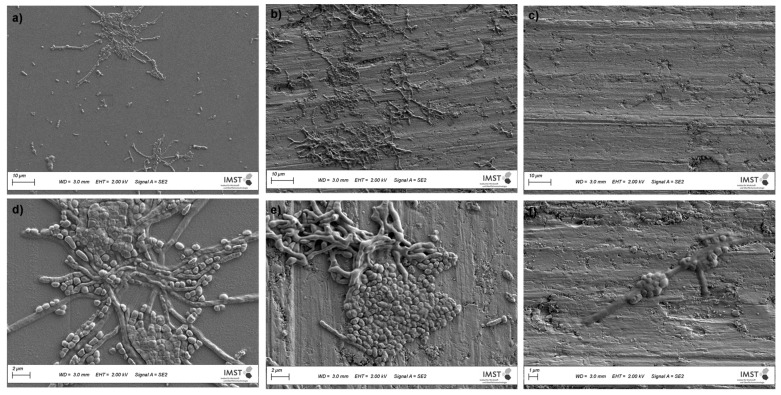
SEM secondary electron micrographs of dental plaque cultures on coverslips (**a**,**d**), as-received TiCP (**b**,**e**), and polySBMA-coated TiCP (**c**,**f**).

As demonstrated in Figure 8c,f, microorganism growth on the polySBMA coatings was scarce, while TiCP (ar) and the coverslips exhibited numerous colony clusters throughout their surfaces. The SEM micrographs shown in Figure 9 depict more detail regarding bacteria proliferation on the treated and as-received specimens. The polySBMA-coated samples show barely any bacteria growth in comparison to the other surfaces, and small isolated colonies were only found after prolonged searching. These colonies are probably associated with coating defects.

## 4. Discussion

Biofilms are known to form on numerous surfaces, with sometimes deleterious and sometimes beneficial effects, depending on the microbial composition [29,30]. The microbiome of the oral cavity is a good example for this issue, where a balanced environment favors symbiosis and oral health, whereas an imbalance creates a dysbiotic state with destructive/corrosive outcomes in regard to biological tissues [31,32]. Far-reaching consequences may be encountered in the context of dental surgery, where a rapidly growing dysbiotic biofilm covering an oral implant surface impairs the surrounding tissue, often resulting in peri-implant diseases [11,33]. Microbial growth on implants is thus of great concern, since most of the widely used implant materials, such as titanium and its alloys, meet the physical, chemical, and tissue biocompatibility properties well but do not prevent microbial fouling [34,35,36,37,38]. To address these issues, various strategies (often inspired by nature) have been suggested [39,40,41,42,43]. Essentially, the main approaches rely on (1) destroying the “intruder” or (2) preventing adhesion. The first approach mainly relies on leachable microbicides, such as silver nanoparticles [44,45,46], antibiotics, anti-microbial peptides [47], nitric oxides, and others [48,49], by way of temporarily impregnating the implant surface. Surface coatings with stable polymer coatings, in some cases together with nano- or micro-patterning, constitute the second approach [19,50,51,52,53]. Moreover, the topographical effects on biofilm formation have been also investigated, and limited favorable effects of roughened and chemically non-modified surfaces have been reported for biofilm formation in comparison to smooth surfaces [37,54].

Surface modification with functional coatings affords a great variety of choices. The resulting coatings can be classified with regard to their chemical nature and physicochemical properties, e.g., hydrophobic, surface hydration, or amphiphilic properties, as well as in terms of their mechanism of action. A great deal of work has been carried out with surface-hydrating coatings, aiming to create surfaces with low interfacial energy with water, which are sometimes called “inert” surfaces [55]. Among others, oligo-, poly(ethylene glycol)-, and zwitterionic acrylates come into focus. Their hydrophilicity, expressed in very low water contact angles, has often been cited and linked to measurable antifouling effects; however, hydrophilicity is not equivalent to antifouling as many hydrophilic surfaces exhibit no antifouling properties, as is the case for glass. What seems to drive the development of non-adhesion properties in the case of hydrophilic surfaces (including superhydrophilic surfaces) is the interaction mode of water molecules at the interface. The greater the H-bonding structure of the interfacial water film resembles that of bulk water, the greater the energetic state of this film favors antifouling, thus preventing the replacement of interfacial water molecules with fouling species [3,7]. This latter point seems to constitute the difference between zwitterionic- and ethylene glycol-based coatings [3,7]. Recent computational studies have confirmed the stronger interaction of zwitterionic polymers with water and consequently better antifouling properties [56]. A secondary outcome with a different interfacial water structure between coating classes includes higher resistance of zwitterionic surfaces towards increasing salt concentrations [3,7,22,23].

Zwitterionic polymer films (2D) and coatings (3D) may be used with various material surfaces and nanoparticles using well-established protocols [2,3,6]. Among such processes, photopolymerization has only received limited interest, despite the fact that it is a relatively simple coating method, commonly implying often short processing times and only requiring a few precursors, e.g., a monomer, initiator, and appropriate solvent [19,57]. As outlined above (see the experimental section), a simple photopolymerization process was developed in this work for the coating of titanium substrates with polySBMA, but this process can be extended to other substrates [19,20]. SBMA was polymerized into a bio-, and hemocompatible polysulfobetaine in the presence of PPD, an initiator substance of certified food grade quality, with water as solvent, in order to minimize cytotoxicity and environmental impacts. A short processing time of a few minutes under mild UV radiation (360 nm) makes the scale-up of the coating process easy. On mildly activated as-received TiCP, the polySBMA coating demonstrated stable hydrophilicity over at least 5 days of dry storage. Furthermore, treatment in an ultrasonic bath for over 3 min did not result in excessive damage of the coatings, and nano-indentation studies revealed good adhesion properties, even at high salt concentrations, which is in good agreement with previous reports regarding the salt stability of zwitterionic coatings [7]. These properties are to be traced on the one hand to the strong bonding between the primed substrate and the coating, and, on the other hand, to the particularly tethered morphology of the nanostructured coating as revealed by the AFM image shown in Figure 2b.

Emphasis in this work has been placed on the interactions of the processed polySBMA coatings with dental plaque microbes and hgF. The non-cytotoxic nature of the coating was demonstrated using XTT-testing and bright field microscopy with hgF in direct and indirect contact with the coated samples. There were no significant differences between the behaviors of the coated TiCP samples in comparison to bare TiCP samples. These results confirm the expectations with regards to the choice of non-toxic precursors well and attest to the efficiency of the polymerization method to achieve quasi precursor-free coatings.

The antifouling investigations were conducted using dental plaque and hgf. What makes dental plaque interesting is that it contains a great number of adherent species. The use of hgF was based on the fact that these cells are easily cultured and are characterized by adherent growth on various substrates and playing prominent roles in the overgrowth of devices such as TADs. The assessment of the interaction of polySBMA films with dental plaque microbes revealed a considerable reduction in biofilm formation on the coated surfaces. A similar observation was made for hgF, where adherent fibroblast cultures on coated surfaces were solely observed in cases of isolated coating damage, such as scratches and non-coated sharp sample edges, etc. Bearing in mind the demonstrated cytocompatibility, these findings can then be explained in terms of the existence of an “energy barrier” [3] that rules out the displacement of water molecules out of the hydrating layer by adherent species (e.g., proteins, microbes, and fibroblasts). The polySBMA coating on an implantable material thus impeded microbial growth and biofilm formation and can be qualified as non-cytotoxic, and, at the same time, precludes the adhesion of fibroblasts. These properties denote the attributes of a cytocompatible anti-biofouling coating. At first glance, this overall assessment may raise questions as to the use of such coatings in implantable devices. There is no doubt regarding the benefits of the antifouling properties described here, but the anti-adhesion effect on anchorage-dependent cell cultures may be a concern. Still, in cases where a high degree of tissue integration is not intended, as is the case for TAD and traumatology implants, polySBMA coatings may be valuable in terms of biofilm reduction and the tunable cell adhesion and proliferation options. This issue may be illustrated on miniature screws as orthodontic TADs. Such a device may be divided in three main parts, namely, the (1) head coupled to the active orthodontic appliance and facing the oral cavity, (2) gingival collar designed to promote tight gingival contact, and finally (3) the thread that ensures endosteal insertion. In some cases, inflammation, mucosal overgrowth, and infections related to the gingival insertion part may occur and delay a successful treatment [14,58,59]. In this case, a biocompatible antifouling coating of the gingival collar that at the same time excludes cell adhesion/overgrowth might be useful. Furthermore, photopolymerization allows for a patterned coating process which enables one to leave areas of sample parts uncoated, for instance, in areas where tissue integration is needed (as for the thread), and coating in other areas, i.e., where antifouling and tight fitting to tissue are required. Ongoing work is devoted to the demonstration of the usefulness of such an approach and will be published in an upcoming report.

## 5. Conclusions

A photografting method has been used to process a zwitterionic polymer polysulfobetaine on O_2_ plasma-activated and methacryl silane-primed titanium substrates using an aqueous solution of the monomer and initiator. Irradiation with mild UV light (360 nm) for a few minutes yielded robust and well-adhering polymer films that resisted sonication and sterilization. The FT-IR spectra show all the vibrations pertaining to polySBMA. High-resolution AFM revealed nanostructured films with nanorope-like morphologies that suggest the presence of tethered polymer brushes. Furthermore, the interfacial adhesion between the film and nanoindenter was greatly affected by soaking in saltwater, demonstrating a four-fold increase in adhesion strength when the films were soaked in 0.51 M NaCl prior to nanoindentation testing in comparison to soaking in water. This result demonstrates that the films are not only resistant to strong ionic solutions but that additionally their adhesion strength is strongly improved by a short treatment in such solutions.

The polySBMA films were characterized by an almost superhydrophilic behavior with water contact angle values ranging between 10 and 15°. Furthermore, the WCAs remained stable after aging for a longer time in a dry laboratory atmosphere.

Following a systematic investigation of the cytotoxicity and anti-adhesive properties, it was demonstrated that the polySBMA films do not affect the cytocompatibility of the titanium substrate, with a viability largely above the cytotoxicity threshold set by ISO 10993-5:2009E. Nevertheless, in contrast to the bare Ti substrate, the polySBMA films drastically reduced the adhesion of fibroblast and plaque microbes, which were shown to be only sporadically present on isolated coating imperfections and non-coated sample edges. On the basis of a survey of the literature, this result is thought to arise from the hydrated surface of the polymer that, energetically speaking, does not afford a favorable energy balance for the non-specific adsorption of proteins and microorganisms. Henceforth, these films may be envisaged for temporary anchorage devices, implants for dentistry, traumatology, stents, etc. As the photografting process used in this work is most suitable for patterning a surface, one may easily endow an implant with coated areas to prevent cellular and bacterial adhesion where necessary, and uncoated areas where implant integration is desirable, thus allowing a multifunctional device to be achieved for a better treatment and healing process.

## Figures and Tables

**Table 1:** Number of samples used in XTT assays.

	Sample Types with Cells			No Cells
Incubation Time (h)	polySBMA Coating	Bare TiCp	Thermanox Control	Empty Insert Blank
24	3	3	3	3
48	20	20	20	20
72	21	21	21	21

**Table 2:** FTIR band assignment of different TiCP sample treatments.

Ti Specimen	Wave Number (cm^−1^)	Band Assignment
As-received TiCP	-	
TiCP with O_2_ plasma treatment	3400	O-H from Ti-OH generated species
	<1000	Ti-O
TiCP with O_2_ plasma-treatment and TMSPMA	3500	SiO-H
	1038	Si-O-Si
	<950	SiO-H/Si-O-Ti
TiCP with with O_2_ plasma treatment and TMSPMA-polySBMA	3400	O-H from physisorbed water
	1725	C=O
	1640	O-H from physisorbed water
	1480/1450	C-H from CH3-N^+^/CH_2_
	1180	S=O asymmetric
	1040	S=O symmetric

**Table 3:** Results of the adhesion testing with a cylindrical flat punch on Ti-polySBMA coatings for two soaking conditions. Saltwater designates a 0.6 M NaCl solution.

Scheme	Soaking Conditions	Max. Load (mN)	Max. Depth (nm)	St. Dev.	Pull-Off Force (mN)	St. Dev.	Pull-Off Stress (kPa)	St. Dev.
Ti-polySBMA	Water	2.0	92.2	23.8	0.1	0.0	13.0	4.0
	Saltwater	2.0	266.8	86.0	0.4	0.2	52.2	26.0

**Table 4:** WCA measurements and their standard deviations (SD).

Treatment	Number of Samples	Mean WCA (°)	SD
As-received TiCP	2	87.3	3.8
TiCP with O_2_ plasma treatment	2	3.5	2.1
TiCP 24 h after O_2_ plasma treatment	2	83.0	18.4
TMSPMA treatment	3	84.1	6.0
PolySBMA coating	17	9.4	3.3
PolySBMA stored for 72 h in H_2_O	7	12.9	10.5
PolySBMA stored when dry for 5 days	8	13.8	4.4

## Data Availability

The data presented in this study are available on request from the corresponding author. The data are not publicly available due to privacy.
